# Intra- and inter-individual variation of BIS-index^® ^and Entropy^® ^during controlled sedation with midazolam/remifentanil and dexmedetomidine/remifentanil in healthy volunteers: an interventional study

**DOI:** 10.1186/cc7723

**Published:** 2009-02-19

**Authors:** Matthias Haenggi, Heidi Ypparila-Wolters, Kathrin Hauser, Claudio Caviezel, Jukka Takala, Ilkka Korhonen, Stephan M Jakob

**Affiliations:** 1 Department of Intensive Care Medicine, Bern University Hospital, Inselspital, and University of Bern, Freiburgstrasse, CH-3010 Bern, Switzerland; 2 VTT Technical Research Centre of Finland, Tekniikankatu 1, Tampere P.O. Box 1300, FI-33101 Tampere, Finland

## Abstract

**Introduction:**

We studied intra-individual and inter-individual variability of two online sedation monitors, BIS^® ^and Entropy^®^, in volunteers under sedation.

**Methods:**

Ten healthy volunteers were sedated in a stepwise manner with doses of either midazolam and remifentanil or dexmedetomidine and remifentanil. One week later the procedure was repeated with the remaining drug combination. The doses were adjusted to achieve three different sedation levels (Ramsay Scores 2, 3 and 4) and controlled by a computer-driven drug-delivery system to maintain stable plasma concentrations of the drugs. At each level of sedation, BIS^® ^and Entropy^® ^(response entropy and state entropy) values were recorded for 20 minutes. Baseline recordings were obtained before the sedative medications were administered.

**Results:**

Both inter-individual and intra-individual variability increased as the sedation level deepened. Entropy^® ^values showed greater variability than BIS^® ^values, and the variability was greater during dexmedetomidine/remifentanil sedation than during midazolam/remifentanil sedation.

**Conclusions:**

The large intra-individual and inter-individual variability of BIS^® ^and Entropy^® ^values in sedated volunteers makes the determination of sedation levels by processed electroencephalogram (EEG) variables impossible. Reports in the literature which draw conclusions based on processed EEG variables obtained from sedated intensive care unit (ICU) patients may be inaccurate due to this variability.

**Trial registration:**

clinicaltrials.gov Nr. NCT00641563.

## Introduction

Pain and anxiety are highly prevalent in critically ill patients in intensive care units (ICUs). Sedation, frequently necessary to maintain patient comfort in ICUs, often has undesirable side effects [1,2]. Strategies to reduce the amount of sedatives used have been shown to improve outcomes [3,4]. To avoid oversedation, sedation levels are assessed, usually by waking the patient regularly and evaluating their responses using a validated scoring system, such as the Ramsay Score (RS) [5], the Sedation-Agitation Scale (SAS) [6] or the Richmond Agitation Sedation Score (RASS) [7]. Although sedation guidelines recommend using a structured assessment system [8], recent surveys demonstrate that less than 50% of ICUs do so [9-11]. Why the tools are not used is unclear, but one reason may be reluctance to awaken patients.

The use of simple, automated, objective, online sedation monitors could help to overcome the shortcomings of the discontinuous and cumbersome sedation scores. Online processed electroencephalogram (EEG) monitors have been developed in recent years, with six systems currently available for intraoperative monitoring. More and more often, these monitors are being used outside of the operating room (OR), to monitor sedation in ICUs, emergency rooms, and radiology and gastroenterology suites [12].

Data on the use of these monitors outside the OR are limited. The most extensively studied device is the BIS^® ^[12]. In the ICU, BIS^® ^has demonstrated mixed results for sedation assessment [13-16]; data for Entropy^® ^are scarce. Despite the lack of validation, the BIS-Index^® ^is routinely used as a sedation goal in some ICUs [12], and its use is advocated by the manufacturer.

The main problem of these devices is the wide inter-individual variation and overlap of the indicated values in lightly sedated patients [17]. Processed EEG values have been compared with clinical sedation scores as the mean value recorded in a definite time epoch, but these time epochs have varied widely between studies, ranging from an average of 10 seconds [15] to one minute before assessment [18], one minute during assessment [14], 15 minutes before assessment [13] and up to an average of two hours before assessment [19]. In other studies, the time epoch is not mentioned at all [16]. If used clinically, the change over time in the individual patient is more relevant. Hence intra-individual variation at specific sedation levels is important. This has not been addressed in previous studies.

We assessed the intra-individual and inter-individual (or within- and between-individual) variability over time of two online sedation monitors – the BIS-Index^® ^and Entropy^® ^– in healthy volunteers during controlled, clinically relevant light sedation with two different sedation regimes.

## Materials and methods

We used data recorded during a study of assessment of sedation levels with long-latency acoustic evoked potentials, also called 'event-related potentials' (ERPs) [20]. Data from the Entropy^® ^Module were not analysed in this previous publication. The study was approved by the ethics committee of the Canton of Bern (KEK Bern), Switzerland, written informed consent was obtained from each individual and the trial was registered at clinicaltrials.gov (Nr. NCT00641563).

In brief, 10 healthy volunteers were sedated in a stepwise manner to achieve RS (Table 1) of 2, 3 and 4 on two occasions separated by one week. In order to maintain constant plasma concentrations, the drugs were given by computer-controlled syringe drivers using the Rugloop II TCI program (BVBA Demed, Temse, Belgium) and published pharmacokinetic and pharmacodynamic datasets [21-23]. Remifentanil was targeted to reach a fixed plasma level of 2 ng/mL in both sessions, and midazolam and dexmedetomidine were titrated to attain the desired sedation levels of RS 2, 3 and 4. The Rugloop II TCI program adjusted the doses to keep the plasma concentrations stable. The predicted mean plasma concentrations based on the actual infusion rates needed to achieve the target sedation levels for dexmedetomidine were 194 ± 17 pg/mL at RS 2, 544 ± 174 pg/mL at RS 3 and 1033 ± 235 pg/mL at RS 4. Those for midazolam were 16 ± 3.7 ng/mL at RS 2, 31 ± 9.6 ng/mL at RS 3 and 56 ± 11.7 ng/mL at RS 4. Assessments of RS were performed by two observers (MH, KH, or CC) right before the recording period and at least 15 minutes after the last drug adjustment to obtain a steady state, and right at the end of the sedation period. If the assessments of the observers differed, consensus was sought.

**Table 1:** The slightly modified Ramsay Score (RS), with a painful stimulus to discriminate between RS 4 and RS 5

Sedation score	Clinical response
1	Fully awake
2	Drowsy, but awakens spontaneously
3	Asleep, but arouses and responds appropriately to simple verbal commands
4	Asleep, unresponsive to commands, but arouses to shoulder tap or loud verbal stimulus
5	Asleep and only responds to firm facial tap and loud verbal stimulus
6	Asleep and unresponsive to both firm facial tap and loud verbal stimulus

At each sedation level, two sets of acoustic stimulation containing short 800 Hz tones with different stimulation presentation were administered by headphones. The stimulation was applied according to both a habituation and a single-tone paradigm. In the habituation paradigm, four equal auditory stimuli were applied through earphones at intervals of one second, followed by a pause of 12 seconds. Altogether, 40 sets of stimuli were delivered at each measurement, corresponding to a recording time of about 10 minutes. In the single-tone paradigm, the same standard tone as described above was delivered 600 times with an interstimulus interval of one second, which also corresponded to a recording time of 10 minutes. The loudness was about 30 dB above the hearing level, but not individually adjusted. During these ERP recording periods, we also registered BIS-Index^® ^(including frontal electromyogram in the 70 to 100 Hz band), state entropy (SE) and response entropy (RE) with the standard BIS^®^-Module (XP-Level, 30-second smoothing time, BIS^®^-Module; GE, Helsinki, Finland) and M-Entropy^® ^Module (GE, Helsinki, Finland) of a Datex-Ohmeda S/5 monitor (GE, Helsinki, Finland), along with other standard monitoring parameters (heart rate/echocardiogram, pulse oximetry, arterial blood pressure via intraarterial catheter and end-tidal CO_2_-concentration via a nasal probe. The processed EEG parameters were recorded online with S/5-Collect software (WinCollect^®^, GE, Helsinki, Finland), and saved on a laptop for further analysis. BIS^® ^values recorded with a Signal Quality Index (SQI) below 50% were not used, as recommended by the manufacturer. Entropy^® ^values were used only when the M-Entropy^® ^built-in data quality control mechanism reported sufficient data quality.

The EEG parameter data were reduced to 10-second intervals, so a maximum of 120 EEG values per patient per sedation level and drug combination could be gathered. As BIS-Index^® ^and Entropy^® ^are proprietary parameters, we assumed they are on rank scales and, therefore, we applied non-parametric statistics for variation.

## Results

**Figure 1:**
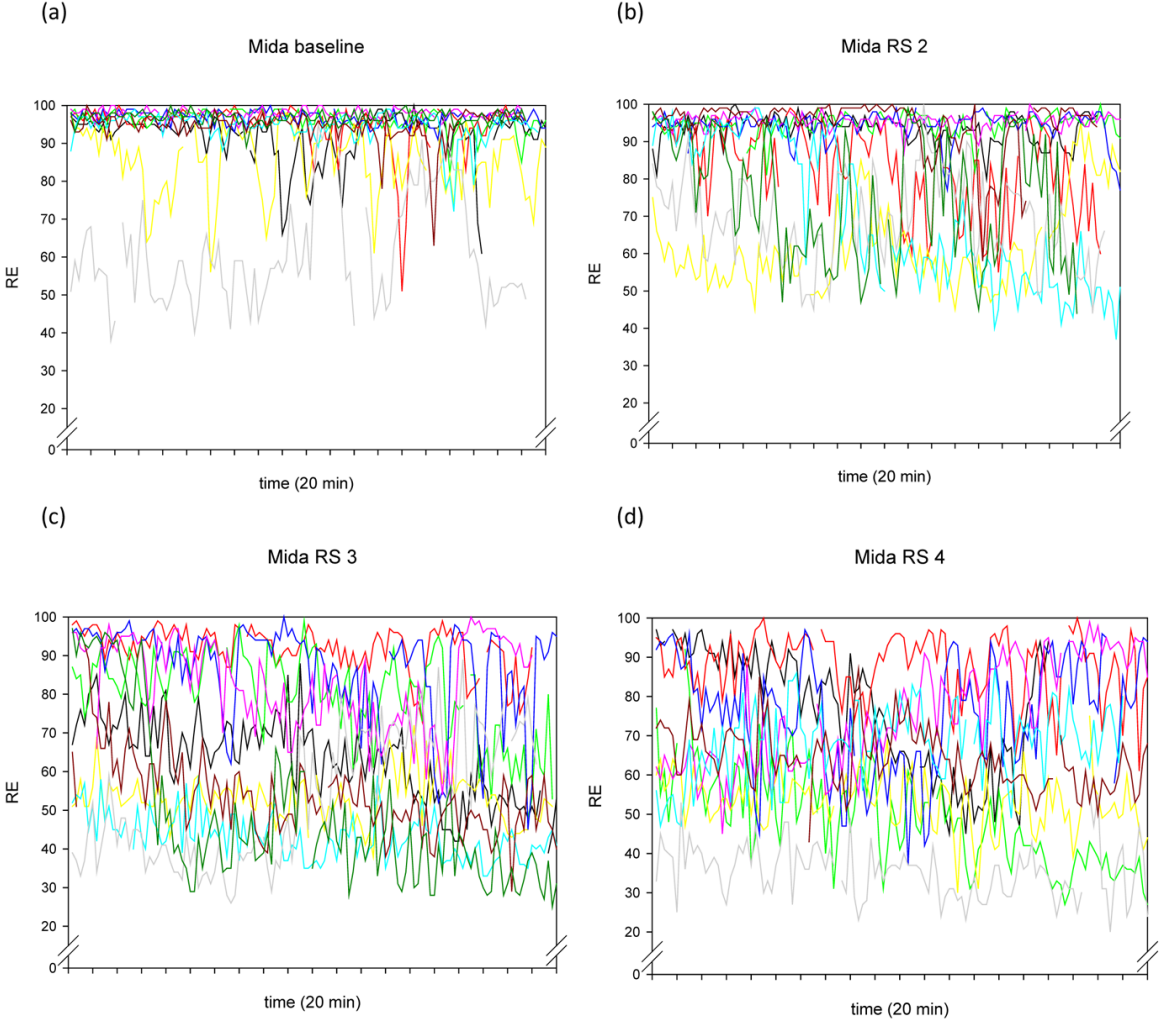
The individual time courses of RE during the 20-minute recordings of the 10 volunteers, at different Ramsay Scores, for the drug combination midazolam/remifentanil. Mida = midazolam/remifentanil; RE = response entropy; RS = Ramsay Score.

**Figure 2:**
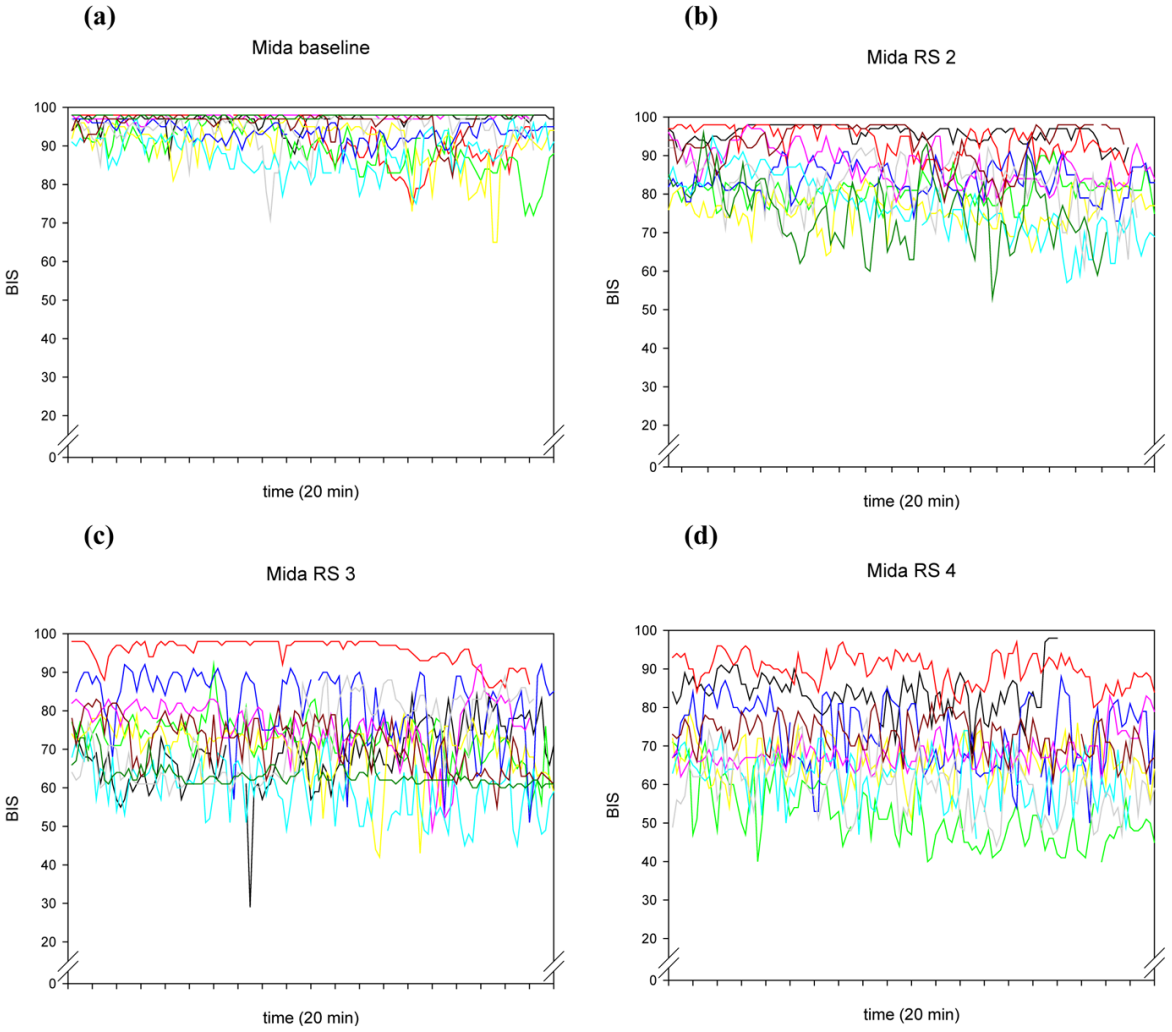
The individual time courses of BIS^® ^during the 20-minute recordings of the 10 volunteers, at different Ramsay Scores, for the drug combination midazolam/remifentanil. Mida = midazolam/remifentanil; RS = Ramsay Score.

**Figure 3:**
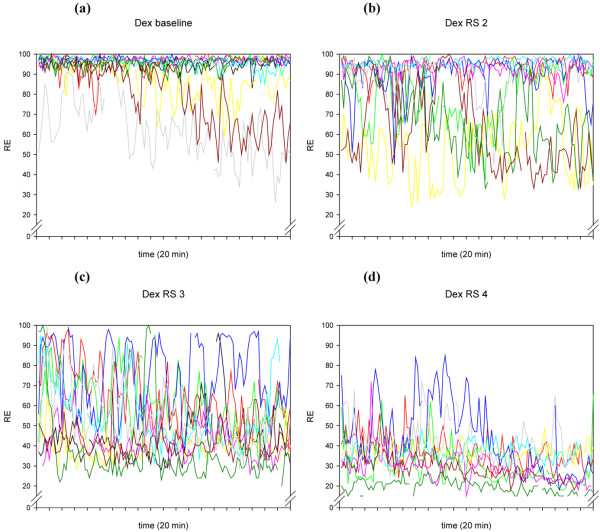
The individual time courses of RE during the 20-minute recordings of the 10 volunteers, at different Ramsay Scores, for the drug combination dexmedetomidine/remifentanil. Dex = dexmedetomidine/remifentanil; RE = response entropy; RS = Ramsay Score.

**Figure 4:**
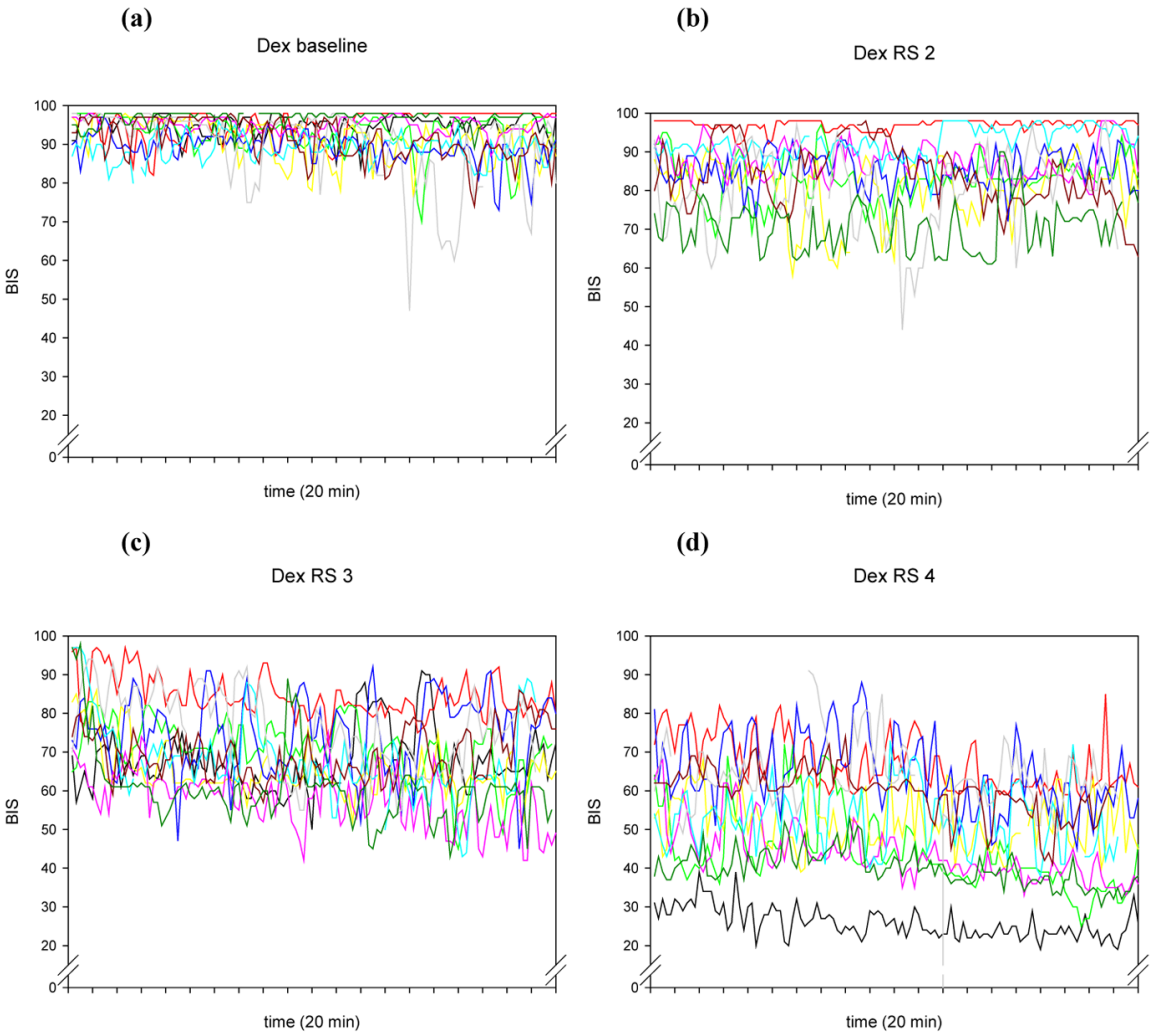
The individual time courses of BIS^® ^during the 20-minute recordings of the 10 volunteers, at different Ramsay Scores, for the drug combination dexmedetomidine/remifentanil. Dex = dexmedetomidine/remifentanil; RE = response entropy; RS = Ramsay Score.

**Figure 5:**
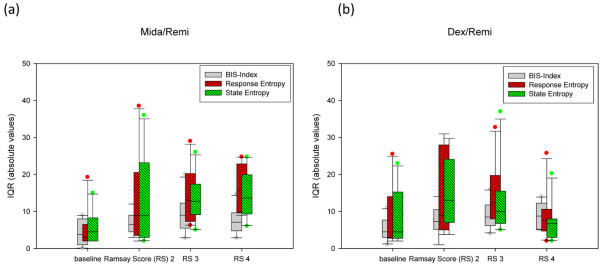
Variation of EEG parameters during the 20-minute recordings in individual patients. Patients received either **(a)** midazolam/remifentanil (Midi/Rem) or **(b)** dexmedetomidine/remifentanil (Dex/Remi). Data are presented as interquartile ranges (IQR), absolute values. EEG = electroencephalography; RS = Ramsay Score.

The individual time courses of BIS-Index^® ^and RE are presented in Figures 1 to 4. In Table 2 we report the inter-individual values for medians and interquartile ranges (IQR) of BIS-Index^®^, RE, SE and frontal electromyography (EMG) from each 20-minute epoch in the 10 volunteers. In addition, we report the range of individual (20-minute epochs) IQRs in Table 2 and Figure 5.

**Table 2:** Inter-individual medians and interquartile ranges and intra-individual ranges of interquartile ranges of each of the 10 patients' individual 20-minute epochs. BIS^®^-Dex/Remi = dexmedetomidine and remifentanil; EMG = electromyogram from BIS^® ^(absolute power in dB (70 to 100 Hz)); IQR = interquartile ranges; Midi/Remi = midazolam and remifentanil; RE = response entropy; SE = state entropy

			**Inter (between)-individual**		**Intra (within)-individual (range of all within variations)**	
					
			Median	IQR	IQR range	Median data points
**Mida/Remi**	Baseline	BIS^®^	94.7	4.25	0 to 9	116.5
		SE	79.9	5.75	2 to 15	115
		RE	91.6	5.35	2 to 19.25	115
		BIS^®^-EMG	44	3.5	2 to 9	116.5
	
	RS 2	BIS^®^	85.0	6.96	3 to 12	117
		SE	73.0	13.35	3 to 36	116
		RE	83.2	13.15	2 to 38.5	116
		BIS^®^-EMG	39	6.0	3 to 10	117
	
	RS 3	BIS^®^	74.4	9.25	4 to 20	117
		SE	57.6	13.35	5 to 26	118
		RE	65.6	14.38	6.25 to 21	118
		BIS^®^-EMG	35	2.1	0 to 5	117
	
	RS 4	BIS^®^	69.0	7.48	2.75 to 14.75	118
		SE	53.9	14.38	6 to 24.75	118
		RE	63.6	16.18	9 to 24.75	118
		BIS^®^-EMG	31	1.6	0 to 4	118

**Dex/Remi**	Baseline	BIS^®^	93.8	5.10	1 to 11	120
		SE	79.9	8.00	2 to 23	118
		RE	91.0	7.75	2 to 25.5	118
		BIS^®^-EMG	51	5.5	2 to 12	120
	
	RS 2	BIS^®^	84.8	7.58	1 to 14	119
		SE	69.8	15.47	4 to 29.75	118
		RE	81.3	16.08	5 to 31	118
		BIS^®^-EMG	34	3.9	2 to 7	119
	
	RS 3	BIS^®^	69.0	9.26	4 to 16	119
		SE	42.9	12.80	5 to 37	118
		RE	49.3	14.98	8 to 32.75	118
		BIS^®^-EMG	32	3.2	0 to 8	119
	
	RS 4	BIS^®^	50.3	8.95	2 to 14	120
		SE	27.3	7.03	2 to 20.25	118
		RE	30.2	8.95	2 to 25.75	118
		BIS^®^-EMG	30	1.6	0 to 5	120

**Figure 6:**
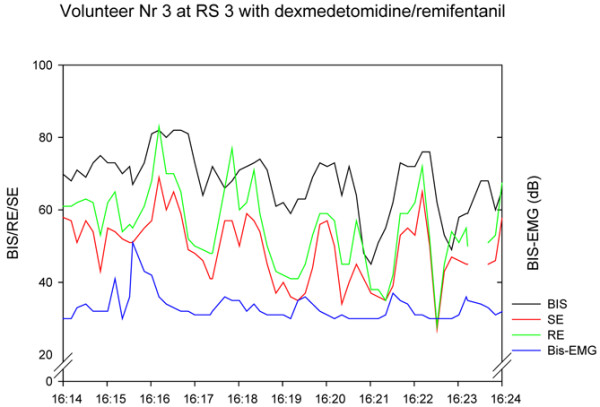
Example of individual variation within a 10-minute recording. The variation is not consistently following frontal muscle electromyogram (EMG). RE = response entropy; RS = Ramsay Score; SE = state entropy.

As expected, the values of the processed EEG decreased as the sedation level increased, with lower BIS^® ^and Entropy^® ^values in the dexmedetomidine/remifentanil group compared with the midazolam/remifentanil group, despite the same sedation levels. The IQRs of BIS^® ^and Entropy^® ^also increased (absolute and relative to the median BIS^®^/Entropy^®^) as sedation levels increased, with Entropy^® ^showing higher variability. The variability was also more pronounced in the dexmedetomidine/remifentanil group than in the midazolam/remifentanil group. Frontal muscle EMG and its variability decreased when sedation increased (Table 2). In Figure 6 we show an example of the variations of the processed EEG and the EMG during a recording at RS 3 with dexmedetomidine/remifentanil.

## Discussion

These data demonstrate wide variation and overlay of the processed EEG data BIS-Index^®^, SE and RE, which increases as the sedation levels decreases, and which also varies depending on the drug combination used. The other concern is high intra-individual variation, up to an IQR of more than 30 in individuals for SE/RE, independent of the drug combination used.

The values of BIS-Index^®^, SE and RE in the midazolam/remifentanil group were in the expected range. More surprising were the extremely low values of the dexmedetomidine/remifentanil group, which have also been reported by other authors [24]. Differences in processed EEG parameters with the use of different drugs can be explained by the drug site of action. Dexmedetomidine binds on α-2 receptors at the locus ceruleus, promoting natural sleep pathways, whereas midazolam (and propofol) potentiate the inhibitory action mediated by the neurotransmitter gamma-aminobutyric acid at the GABA A receptor [25]. It is well known that different drugs induce different EEG patterns at the same anaesthetic point [26], so the differences in the EEG pattern of the drug can simply be a drug effect, as described with other drugs such as ketamine, which also fail to follow the usual pattern of the BIS^® ^[12].

High variation of Entropy^® ^has also been described in patients in whom SE and RE followed an unpredictable on/off pattern [18]. The authors attributed this to variation of EMG activity and resulting EMG power, and also in frequency ranges which are not affected by frontal muscle activation in deep anaesthesia. Our results demonstrate that variability of EMG activity does not explain the variability of processed EEG parameters. We suggest another possible explanation: underlying oscillatory systems [27], also known as 'sleep spindles' or 'propofol spindles', with their regular patterns, are almost perfect sinus curves, and therefore have low entropy, translating into a low M-Entropy^® ^value when they appear. We also intermittently observed large, regular waves at 3 Hz frequency with resulting low Entropy^® ^values in volunteers receiving the dexmedetomidine/remifentanil combination, which accounts in part for the high intra-individual variation, particularly seen in the dexmedetomidine/remifentanil groups.

A more general explanation for this high within-individual variability is offered in research performed by Lu and colleagues [28]. These authors describe several positive feedback neuronal mechanisms of the brain, tending to force the brain to be either fully awake or fully asleep. These mechanisms are activated by both alpha-adrenergic pathways and GABAergic inputs. The traces in Figures 1 to 4 seem to show several subjects jumping between EEG states, presumably related to internal or external stimuli. Furthermore, the traces seem to track a subset of people who become sedated but retain high frequencies in their EEG, and therefore a high RE (particularly) or BIS^®^. This might be a state analogous to rapid eye movement (REM) sleep.

Auditory stimuli applied during measurement of sedation may theoretically influence the sedation level. Absalom and colleagues tested this hypothesis and did not find a clinically significant effect of auditory stimuli on BIS-Index^® ^[29]. Also, the relatively long duration of the recordings (20 minutes) can be criticised, because in those 20 minutes volunteers can both fall asleep (causing slowing of EEG activity) and be aroused by external stimuli (such as ICU alarms). In any case, some variations in EEG parameters should be expected, particularly when dexmedetomidine is used, because this drug is known to produce arousable sedation which will naturally be accompanied by EEG activation [30].

Despite all these potential confounders, the clinical sedation status of the volunteers as observed by the research personnel did not change during the recording time. We consider these points to be a strength rather than a weakness of the study because our approach represents real-life conditions encountered by ICU patients. If low variability of processed EEG was observed in a quiet laboratory, the 'unreal' surrounding would certainly have been criticised. Nevertheless, our results cannot necessarily be extrapolated to ICU patients because of the complex interactions between different drugs and diminished organ functions, sometimes including encephalopathy and delirium. These factors are likely to increase variability even more.

## Conclusion

When physiological variables are used to support clinical decision-making, trends rather than absolute individual values are relevant. The overlap of values representing different clinically relevant sedation levels, as well as the high intra-individual variability, especially in Entropy^®^, calls into question even the use of trends in these variables to support clinical decisions or as therapeutic targets.

## Key messages

- BIS-Index^® ^and Entropy^® ^values in the dexmedetomidine/remifentanil group are lower compared with the midazolam/remifentanil group, despite the same sedation levels.

- Variability of BIS-Index^® ^and Entropy^® ^increase (absolute and relative to the median BIS^®^/Entropy^®^) as sedation deepens.

- Entropy^® ^shows a higher variability compared with BIS-Index^®^.

- Variability is more pronounced in the dexmedetomidine/remifentanil group than in the midazolam/remifentanil group.

- BIS-Index^® ^and Entropy^® ^values in sedated volunteers are not determined by sedation levels.

## Abbreviations

EEG: electroencephalogram; EMG: electromyography; ERP: event-related potential; ICU: intensive care unit; IQR: interquartile range; OR: operating room; RASS: Richmond Agitation Sedation Score; RE: response entropy; REM: rapid eye movement; RS: Ramsay Score; SAS: Sedation-Agitation Scale; SE: state entropy; SQI: Signal Quality Index.

## Competing interests

The study was funded by an unrestricted grant from Instrumentarium/Datex-Ohmeda, now GE Healthcare, Helsinki, Finland. The study design was approved, but not influenced by GE Healthcare. Instrumentarium/Datex-Ohmeda was not involved in any way in collection, analysis and interpretation of data, in writing of the manuscript or in the decision to submit this manuscript.

MH, KH, CC, JT and SMJ: The Department of Intensive Care Medicine has received research funding from GE Healthcare to carry out research projects related to the depth of anaesthesia monitoring. A part of the work reported here has resulted from these projects.

HY and IK: VTT Technical Research Centre of Finland has received funding from GE Healthcare to carry out research projects related to the depth of anaesthesia monitoring. Both authors have been working in these research projects, and part of the work reported here has resulted from these projects.

## Authors' contributions

MH conceived and designed the study, contributed to acquisition, analysis and interpretation of data, performed the statistical analysis and drafted the manuscript. HY made substantial contributions to data acquisition and interpretation. KH and CC planned the study and collected and analyzed the data. JT contributed to study design, data interpretation and drafting the manuscript. IK contributed to data analysis and revised the manuscript. SMJ conceived the study, and contributed substantially in all parts of the study and manuscript preparation. All authors have given final approval of the version to be published.
